# Premature contractions of the bladder are suppressed by interactions between TRPV4 and SK3 channels in murine detrusor PDGFRα^+^ cells

**DOI:** 10.1038/s41598-017-12561-7

**Published:** 2017-09-25

**Authors:** Haeyeong Lee, Byoung H. Koh, Lauren E. Peri, Robert D. Corrigan, Hyun-Tai Lee, Nikita E. George, Bhupal P. Bhetwal, Yeming Xie, Brian A. Perrino, Toby C. Chai, Kenton M. Sanders, Sang Don Koh

**Affiliations:** 1 Department of Physiology and Cell Biology, University of Nevada School of Medicine, Reno, NV 89557 USA; 2 Department of Urology, Yale University School of Medicine, New Haven, CT 06519 USA

## Abstract

During filling, urinary bladder volume increases dramatically with little change in pressure. This is accomplished by suppressing contractions of the detrusor muscle that lines the bladder wall. Mechanisms responsible for regulating detrusor contraction during filling are poorly understood. Here we describe a novel pathway to stabilize detrusor excitability involving platelet-derived growth factor receptor-α positive (PDGFRα^+^) interstitial cells. PDGFRα^+^ cells express small conductance Ca^2+^-activated K^+^ (SK) and TRPV4 channels. We found that Ca^2+^ entry through mechanosensitive TRPV4 channels during bladder filling stabilizes detrusor excitability. GSK1016790A (GSK), a TRPV4 channel agonist, activated a non-selective cation conductance that coupled to activation of SK channels. GSK induced hyperpolarization of PDGFRα^+^ cells and decreased detrusor contractions. Contractions were also inhibited by activation of SK channels. Blockers of TRPV4 or SK channels inhibited currents activated by GSK and increased detrusor contractions. TRPV4 and SK channel blockers also increased contractions of intact bladders during filling. Similar enhancement of contractions occurred in bladders of *Trpv4* ^−/−^ mice during filling. An SK channel activator (SKA-31) decreased contractions during filling, and rescued the overactivity of *Trpv4* ^−/−^ bladders. Our findings demonstrate how Ca^2+^ influx through TRPV4 channels can activate SK channels in PDGFRα^+^ cells and prevent bladder overactivity during filling.

## Introduction

Overactive bladder is a clinical syndrome accounting for over $12 billion in health care costs in the USA annually^1^. Current therapies to control this problem are less than adequate^2^. How the bladder remains mechanically stable during filling is a question lacking clear physiological explanation. We recently described a new class of interstitial cells in the murine bladder that are labeled specifically with antibodies against platelet-derived growth factor receptor-α (PDGFRα^+^ cells)^3^. We also identified a reporter strain of mice in which PDGFRα^+^ cells express a histone 2B-eGFP fusion protein driven off the endogenous promoters for *Pdgfra* (Pdgfrα/eGFP mouse)^3^. PDGFRα^+^ cells have also been identified in human and guinea pig detrusor muscles^4^. These cells are closely associated with varicose nerve processes in detrusor muscles (*muscularis propria*) suggesting that PDGFRα^+^ cells may be innervated and participate in purinergic regulation of bladder contraction^5–7^.

PDGFRα^+^ cells in detrusor muscles express small conductance Ca^2+^-activated K^+^ (SK3) channels^5^, and it is known that SK channel blockers and genetic deactivation of SK3 evokes bursts of action potentials in detrusor muscle preparations and increase contractions in response to stimulation of motor neurons^8–11^. PDGFRα^+^ cells display high current densities due to SK channels (i.e. whole cell currents averaging 13 pA/pF at −40 mV), but detrusor smooth muscle cells (SMCs) show very low SK current density (e.g. 0.5 pA/pF at + 10 mV)^5^. Thus stabilization of bladder excitability by SK channels appears to be due to the prominent expression of these channels in PDGFRα^+^ cells rather than in SMCs.

SK channels are activated by increased intracellular Ca^2+^ ^12,13^. In many cells Ca^2+^ influx occurs through activation of voltage-dependent Ca^2+^ channels. However, functional voltage-dependent Ca^2+^ conductances have not been resolved in PDGFRα^+^ cells^5^, raising the question of the source of Ca^2+^ for the regulation of SK channels in these cells. Transient receptor potential (TRP) channels are expressed by most cells and vanilloid TRP (TRPV) channels are relatively permeable to Ca^2+^ ^14^. Previous studies have shown *Trpv4* is expressed in extracts of whole detrusor muscles that would have contained transcripts from SMCs and PDGFRα^+^ cells^15^. *Trpv4* ^−/−^ mice displayed increased non-voiding contractions^16,17^. We tested the hypothesis that TRPV4 channels are expressed primarily by PDGFRα^+^ cells in detrusor muscles, and that Ca^2+^ entry via TRPV4 channels is linked to activation of SK channels. It is also important to note that TRPV4 channels can be mechanically activated^14,18^. Thus, it is possible that enhanced Ca^2+^ influx via TRPV4 channels during bladder filling increases the open probability of SK channels to dampen the excitability of detrusor muscles. Our results show that SK channel activation is closely linked to TRPV4-mediated Ca^2+^-influx and may be enhanced by direct interactions between TRPV4 and SK3 channels in PDGFRα^+^ cells.

## Results

### Expression of Trpv4 in PDGFRα^+^ cells

**Figure 1:**
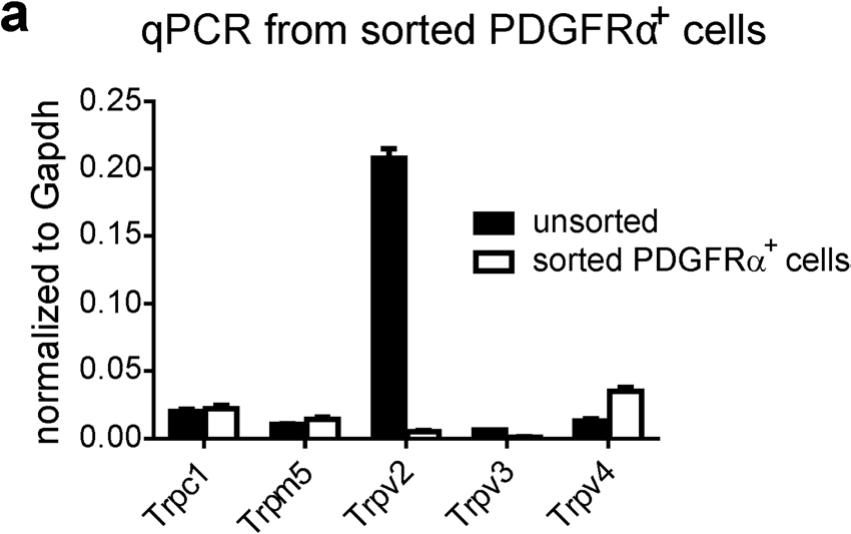
Quantitative analysis of *Trp* transcripts from sorted Pdgfrα^+^ cells. Quantitative analysis of *Trp* transcripts revealed *Trpv4* is highly expressed in sorted PDGFRα^+^ cells (n = 4).

We investigated the expression of *Trp* genes in PDGFRα^+^ cells. We have shown previously that cells isolated enzymatically from bladders of *Pdgfra* ^*tm11*(*EGFP*)*S*^ *°* ^*r*^/J mice and sorted by fluorescence activated cell sorting (FACS) display significant enrichment in *Pdgfrα* transcripts and negligible expression of *kit* (ICC marker), *Uchl1* (neuronal marker) and *Myh11* (SMC marker)^5^. We isolated PDGFRα^+^ cells from detrusor muscles, purified these cells by FACS, and probed for expression of *Trp* genes. We found expression of *Trpc1*, *Trpm5*, *Trpv2*, *Trpv3* and *Trpv4* in PDGFRα^+^ cells. *Trpc2*, *Trpc6*, *Trpm7 and Trpv2* transcripts were detected in SMCs (obtained from smMHC/Cre/eGFP mice; data not shown). Quantitative analysis of *Trp* transcripts from PDGFRα^+^ cells showed that *Trpv4* (2.7 ± 0.2 fold) was highly expressed in PDGFRα^+^ cells vs. unsorted cells of the detrusor (n = 4, Fig. 1). Thus, we focused our investigations on the functional role of TRPV4 channels in PDGFRα^+^ cells since TRPC1 and TRPM5 channels are less permeable to divalent cations.

### Effects of TRPV4 agonist and antagonists on PDGFRα^+^ cells

**Figure 2:**
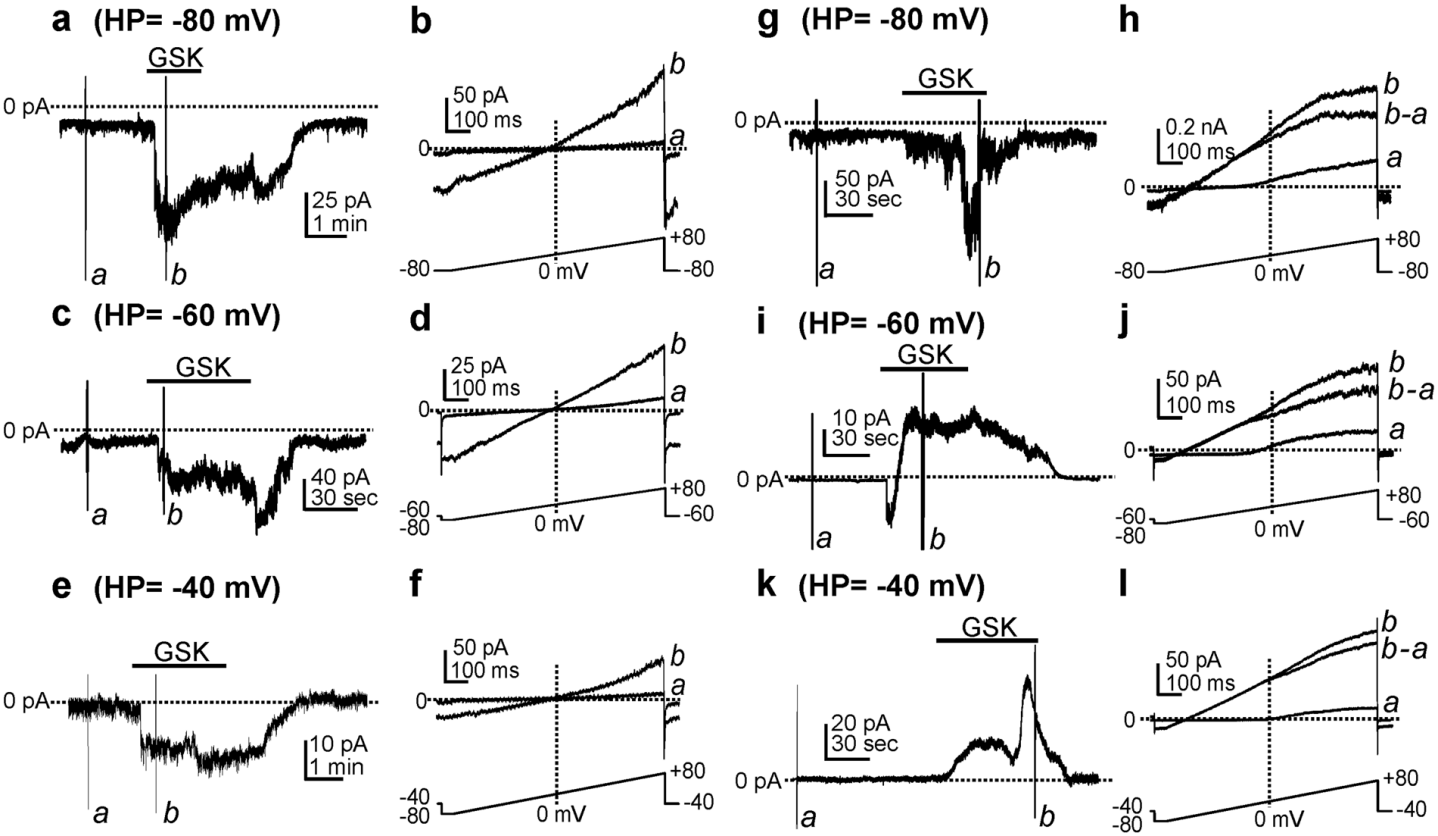
The effect of TRPV4 agonist on the inward currents and the outward currents in PDGFRα^+^ cells. GSK1016790A (GSK, 100 nM) activated inward currents at holding potentials −80 to −40 mV. Cells were dialyzed with Cs^+^-rich solutions (**a**,**c**,**e**). Expanded time scales (**b**,**d**,**f**) from each left panel during ramp depolarization at different holding potentials. *a* & *b* denote before and after GSK (100 nM), respectively. When cells were dialyzed with K^+^-rich solutions, GSK (100 nM) activated inward current at a holding potential of −80 mV (**g**). Expanded time scales (**h**) from panel **g** during ramp depolarization before (*a*) and after (*b*) GSK (100 nM) application, respectively. *b*-*a* denotes GSK-sensitive current. GSK (100 nM) activated inward current followed by outward current at holding potentials of −60 mV (**i**) and −40 mV (**k**). Expanded time scales (**j**,**l**) from panels i and k during ramp depolarization before (*a*) and after (*b*) GSK application, respectively. *b*-*a* denotes GSK-sensitive current.

We tested the effects of TRPV4 agonist GSK1016790A (GSK)^15^ and antagonists on the generation of membrane currents and potentials in PDGFRα^+^ cells. Under whole-cell patch clamp conditions (cells dialyzed with Cs^+^-rich pipette solution; see Methods), GSK (100 nM) induced inward currents at holding potentials from −80, −60 and −40 mV (Fig. 2a,c,e; n = 12). When cells were depolarized with ramp protocols from −80 mV to + 80 mV (lower inset in Fig. 2b,d,f), negligible currents were evoked in control conditions (Fig. 2b*a*,d*a*,f*a*). GSK activated non-selective cation currents with a reversal potential of −1.0 ± 0.4 mV (Fig. 2b*b*,d*b*,f*b*). When cells were dialyzed with K^+^-rich pipette solution (see Methods), GSK (100 nM) activated inward current at a holding potential of −80 mV (n = 6, Fig. 2g), inward currents followed by outward current at −60 mV (n = 8, Fig. 2i), and outward currents at −40 mV (n = 35, Fig. 2k). The GSK-induced outward currents were voltage-independent and the whole cell reversal potential shifted toward the equilibrium potential for the K^+^ gradient (*E*_K_ = ~−80 mV) (Fig. 2h,j,l).

TRPV4 channels can be activated by 4α-Phorbol 12,13-didecanoate (4α-PDD), swelling and mechanical stretch^19–22^. We examined whether activation of TRPV4 channels in PDGFRα^+^ cells by these alternative methods also led to activation of outward current. Cells were stretched using two patch electrodes: one to measure whole cell current and the other to elongate the cell^23^. After obtaining whole cell conditions with the first electrode, a second gigaseal was formed with the second electrode, and this was used to slowly stretch the cell by 1-2 µm. Mechanical stretch induced transient inward current followed by outward current (supplementary Fig. 2a,b). These effects were similar to the effects of GSK. In another series of experiments hypo-osmotic solution (200 mOsm) was used to swell cells. Exposure to hypo-osmotic solution induced inward current followed by reversal of the response to outward current (supplementary Fig. 2c,d). Finally, we also tested the effects of 4α-PDD, a non-selective TRPV4 agonist. Application of 4α-PDD induced inward current followed by outward current (supplementary Fig. 2e,f). Thus, all methods used to activate TRPV4 current (inward) resulted in secondary activation of an outward current as observed with GSK.

**Figure 3:**
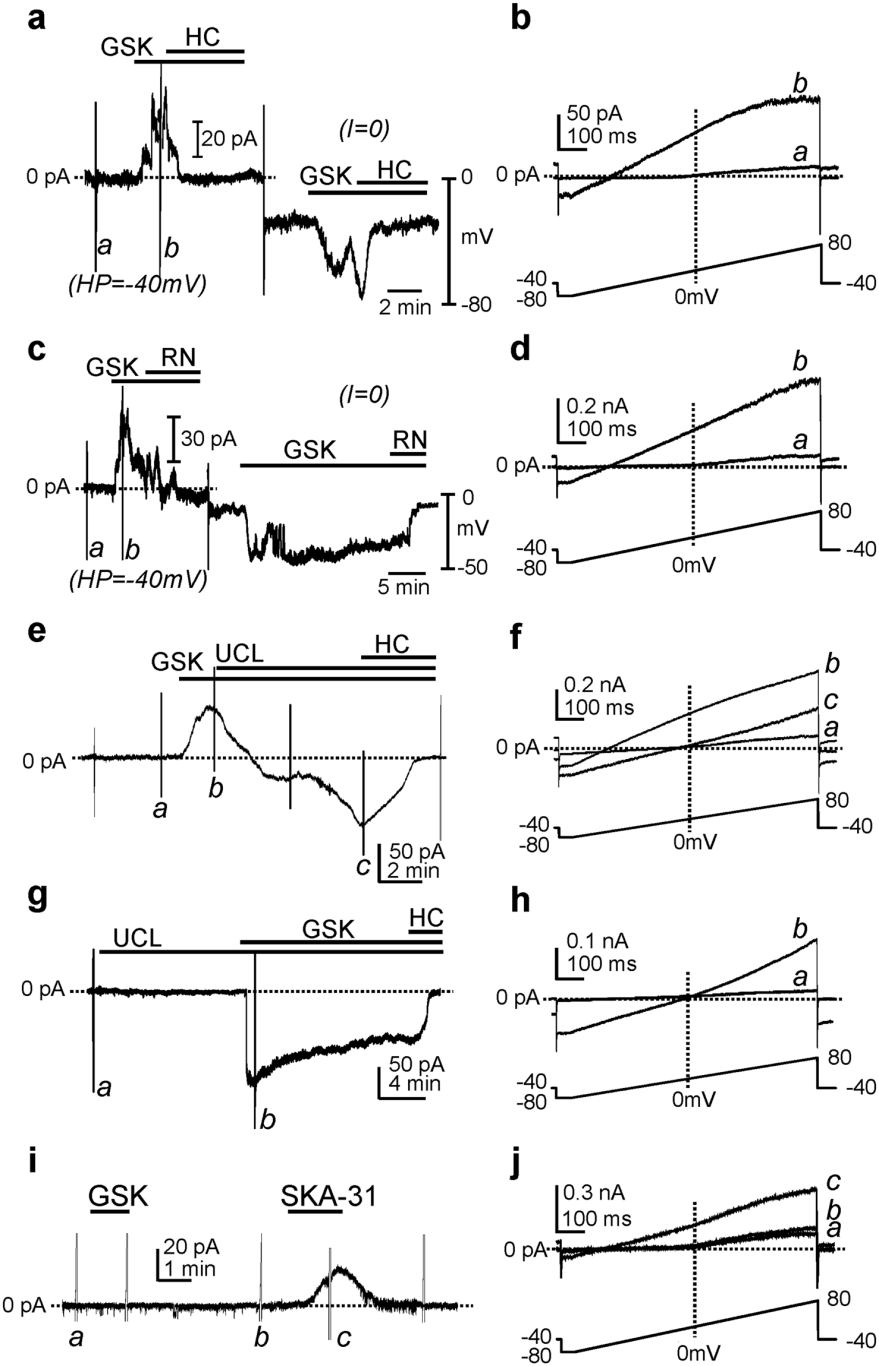
The effect of TRPV4 antagonist and SK channel blocker on the outward currents and membrane potentials in PDGFRα^+^ cells. In cells dialyzed with K^+^-rich solutions, GSK (100 nM) activated outward current at a holding potential of −40 mV in voltage clamp mode. In the same cell, GSK induced hyperpolarization under current clamp (*I* = *0*). TRPV4 antagonist, HC-067047 (1 μM, HC, **a**) and RN-1734 (10 μM, RN, **c**) inhibited GSK-activated outward current and hyperpolarization. Expanded time scales (**b**,**d**) from panels a and c during ramp depolarization before (*a*) and after (*b*) GSK application, respectively. In next experiments, cells were dialyzed with K^+^-rich solutions and held at −40 mV. GSK (100 nM) activated outward current. Addition of an SK channel blocker, UCL1684 (UCL, 1 μM) inhibited the outward currents and revealed an inward current. TRPV4 antagonist, HC-067047 (1 μM, HC) inhibited the inward current activated by GSK (**e**). Expanded time scales (**f**) from panel e during ramp depolarization (see inset) before (*a*), after (*b*) GSK and additional application of HC (*c*). Under pretreatment of UCL, GSK activated only inward current at a holding potential of −40 mV. Addition of HC completely blocked the inward current (**g**). Expanded time scales (**h**) from panel g during ramp depolarization before (*a*) and after (*b*) GSK application in the presence of UCL. PDGFRα^+^ cells from *Trpv4* ^−/−^ mice were dialyzed with K^+^-rich solutions (**i**). GSK (100 nM) did not activate outward current at a holding potential of −40 mV. In the same cell, an SK channel activator (SKA-31, 10 µM) activated outward currents (**i**). Expanded time scales (**j**) from panel **i** during ramp depolarization in control (*a*), and before (*b*) and after (*c*) SKA-31 application, respectively.

A selective TRPV4 antagonist, HC-067047 (1 μM, Fig. 3a,b)^24^ completely abolished the voltage-independent outward current evoked by GSK at −40 mV. In the same cells under current clamp (*I* = 0), GSK caused hyperpolarization averaging −38 ± 5 mV (n = 9). The average resting membrane potential was −35 ± 5.1 mV in control. HC-067047 (1 µM) abolished the GSK-induced hyperpolarization. Another selective TRPV4 antagonist, RN-1734^25^ (10 μM, Fig. 3c,d) also inhibited GSK-activated currents under voltage clamp and abolished GSK-induced hyperpolarization under current clamp (*I* = *0*, n = 7). These data suggest that activation of TRPV4 may result in Ca^2+^ influx and secondary activation of a Ca^2+^-sensitive K^+^ conductance.

SK3 channels are highly expressed in PDGFRα^+^ cells^5^. The outward current activated by GSK had properties consistent with currents mediated by SK channels (i.e. voltage-independent; rectification at positive potentials^5^; see Fig. 2h,j,l). Therefore, we tested the effects of an SK channel blocker, UCL1684^26–28^ on the outward current activated by GSK. Using K^+^-rich internal solution, cells were held at −40 mV. GSK activated outward current (Fig. 3e) and caused a negative shift in the reversal potential (Fig. 3f*b*). UCL1684 (1 µM) blocked the outward current activated by GSK and unmasked the inward current activated by GSK (172 ± 69 pA, n = 6, Fig. 3e) and shifted reversal potentials close to 0 mV (Fig. 3f*c*). The underlying inward current was completely blocked by HC-067047. We also tested the effects of GSK after pretreatment with UCL1684 (1 µM). GSK in the presence of UCL1684 (1 μM) activated inward current (122 ± 72 pA, n = 6), but no outward current, at a holding potential of −40 mV. The inward current reversed at −1.9 ± 0.6 mV and was blocked by HC-067047 (1 μM) (Fig. 3g,h; n = 5). These data suggest that Ca^2+^ influx via TRPV4 channels is sufficient to activate SK current and induce hyperpolarization of PDGFRα^+^ cells.

As a control for the effects of GSK, we tested the effects of this compound on PDGFRα^+^ cells from detrusor muscles of animals obtained by crossing PDGFRα/eGFP and *Trpv4* ^−/−^ mice. GSK failed to activate inward or outward currents in these cells, but the SK channel activator (SKA-31) induced outward current (Fig. 3i,j). This observation suggests that GSK is selective for TRPV4 channels, and these channels are linked to activation of SK channels in detrusor PDGFRα^+^ cells.

### Ca^2+^ influx through TRPV4 channels in PDGFRα^+^ cells

**Figure 4:**
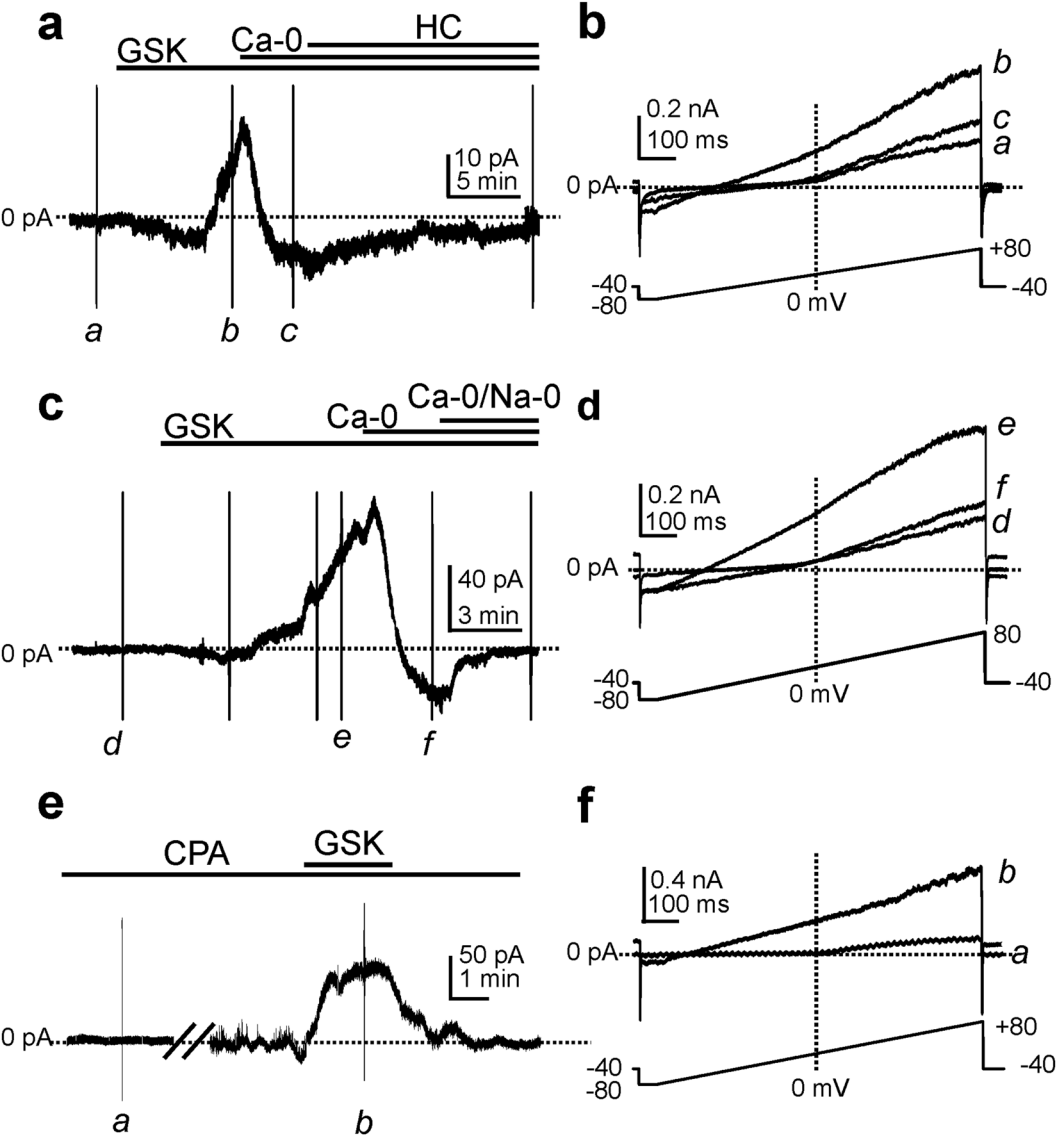
The effects of cation replacement on the GSK-activated currents in PDGFRα^+^ cells. Cells were dialyzed with K^+^-rich solutions at a holding potential of −50 mV. GSK (100 nM) activated a small inward current followed by an outward current. Removal of external Ca^2+^ (Ca-0) inhibited the outward current and unmasked the inward current. Addition of HC-067047 (1 μM, HC) blocked the inward current activated by GSK (**a**). Expanded time scales (**b**) from panel a during ramp depolarization (see inset) before (*a*), after (*b*) GSK and Ca^2+^-free condition (*c*). GSK activated a small inward current followed by an outward current at a holding potential of −50 mV. External Ca^2+^-free solution (Ca-0) blocked the outward current and unmasked the inward current. Addition of external Ca^2+^ and Na^+^ free (Ca-0/Na-0) solution abolished the inward current (**c**). Expanded time scales (**d**) from panel c during ramp depolarization before (*d*), after (*e*) GSK and under Ca^2+^-free condition (*f*). After 10 min incubation with cyclopiazonic acid (CPA, 10 µM) GSK activated outward current at a holding potential of −40 mV (**e**). Expanded time scales (**f**) from panel **e** before (*a*) and after (*b*) GSK in the presence of CPA.

TRPV4 is a non-selective cation channel permeable to Ca^2+^, Mg^2+^, Na^+^ and K^+^^29^. In further tests of whether Ca^2+^ influx through TRPV4 can trigger SK channel currents, we utilized ion replacements. GSK activated small amplitude inward currents followed by outward current at a holding potential of −50 mV (Fig. 4a,c). When external Ca^2+^ was replaced by Mn^2+^ (Ca^2+^−0), the outward phase of the response to GSK disappeared leaving only a small amplitude inward current (Fig. 4a,b). The inward current was inhibited by HC-067047 (Fig. 4a). TRPV4 channels are also permeable to Na^+^. When external Na^+^ was replaced with equimolar NMDG and 0 mM Ca^2+^ (Ca^2+^−0/Na^+^−0), the inward current activated by GSK was abolished (Fig. 4c,d).

It is possible that Ca^2+^ influx via TRPV4 is amplified by Ca^2+^ release from intracellular stores. Experiments were performed on cells dialyzed with BAPTA (10 mM) in the presence of cyclopiazonic acid (10 µM) to test the role of Ca^2+^ stores in GSK responses. Under these conditions, GSK still activated outward currents (Fig. 4e,f), suggesting that Ca^2+^ influx through TRPV4 channels directly activates SK channels without initiating intracellular Ca^2+^ signaling (e.g by Ca^2+^ induced Ca^2+^ release).

### TRPV4 current in smooth muscle cells

We also tested whether smooth muscle cells (SMCs) display functional expression of TRPV4, even though *Trpv4* transcripts were not resolved in these cells (not shown). SMCs displayed voltage-dependent inward current during ramp depolarization when cells were dialyzed with Cs^+^-rich solution (supplementary Fig. 1a,b). GSK (100 nM) failed to evoke current responses in SMCs (n = 10). The effects of GSK were also tested on membrane potentials using K^+^-rich internal solution. GSK had no effect on membrane potential (supplementary Fig. 1c,d). These results are consistent with the transcript expression data, and demonstrate no role for TRPV4 channels in detrusor SMCs.

### The effects of TRPV4 agonist and antagonist on detrusor muscle strips

**Figure 5:**
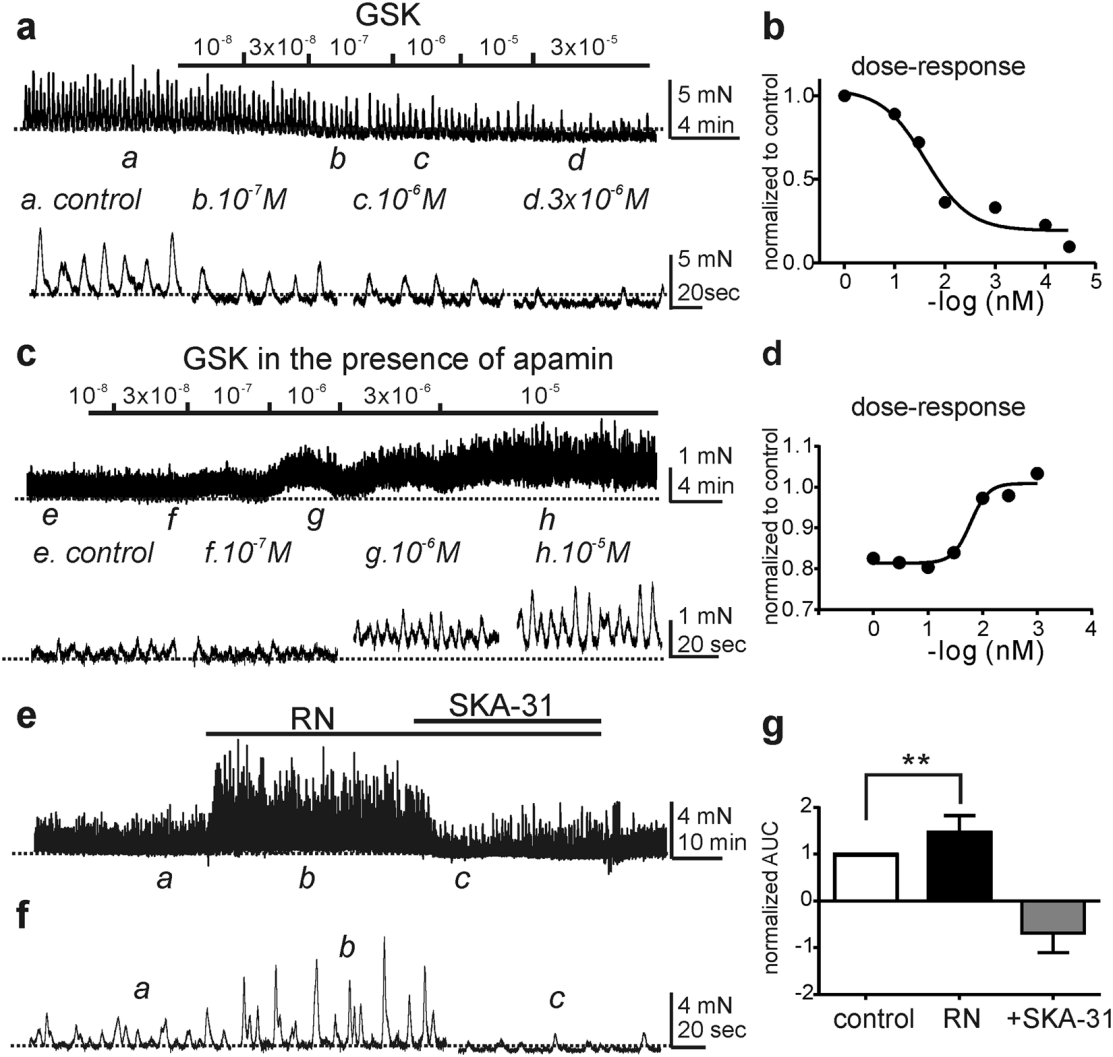
The effects of TRPV4 agonist, antagonist and SK channel activator on phasic contractions in murine bladder strips. Concentration-response relationship for GSK shows inhibitory effect of this TRPV4 agonist on phasic contractions (a, upper panel). Excerpts in the record denoted by *a*–*d* are shown below and show contractions in the presence of 10^−7^, 10^−6^ and 3 × 10^−6^ M GSK (lower panel). Average concentration-response curve for GSK effects with contractile amplitudes normalized as a percentage of control (n = 37, **b**). Representative concentration-response showed that GSK increased the phasic contraction in the presence of apamin (**c**, upper panel) and expanded time scales (lower panel). Average concentration-response curve for GSK in the presence of apamin with contraction amplitudes normalized as a percentage of control (n = 9, **d**). Representative phasic contractions response to RN-1734 (RN, TRPV4 antagonist) and SKA-31 (SKA activator, **e**). Expanded time scales from panel f under control (a), RN-1734 (**b**) and SKA-31 (**c**). Summary of normalized area under curve (AUC) of phasic contractions under control, RN-1734 and SKA-31 (n = 7. **g**). **P < 0.01 and horizontal dotted lines in all panels denote baseline in control.

It was previously reported that the TRPV4 agonist (GSK) increased spontaneous contractions of detrusor muscles with or without urothelium^15^. This result is contrary to our findings, because GSK, through activation of SK channels, would tend to exert inhibitory effects on contractions through activation of SK channels (see Figs 1–4). Therefore, we tested the effects of GSK (10 nM–30 µM) on contractions of detrusor muscle strips with urothelium removed. As predicted, GSK decreased the amplitude and frequency of spontaneous contractions and reduced basal tone in a concentration-dependent manner (Fig. 5a,b, n = 37 of 44 from 14 animals). The IC_50_ for the reduction in amplitude was 41.5 nM. A small number (7 of 44; 16%) of detrusor muscles showed a small increase in contractions at higher concentrations of GSK (≥1 µM). The TRPV4 agonist activated SK channels in PDGFRα^+^ cells, so the relaxation caused by the TRPV4 agonist might be due to activation of SK channels. To investigate this hypothesis, detrusor muscle strips were pretreated with apamin (Fig. 5c,d). In the presence of apamin GSK caused a concentration-dependent increase in contractions with an EC_50_ of 770 nM (Fig. 5d). The effects of a TRPV4 antagonist (RN-1734) on the contractility of detrusor muscle strips were also examined. RN-1734 (10 µM) increased contractions (area under curve; AUC) to 148 ± 13% compared to control (n = 7, *P* < 0.01, Fig. 5e–g). This observation is consistent with a link between TRPV4 channels and activation of SK channels. SKA-31 (10 µM, SK channel activator) reduced contractions caused by RN-1734 to 67 ± 15% of control (Fig. 5e–g). These findings suggest that the mechanical stretch (1 mN) applied to muscles under basal condition, enhanced activation of TRPV4 channels and produced basal activation of SK channels.

### The effects of TRPV4 agonist and antagonist on *ex vivo* pressure-volume measurement

**Figure 6:**
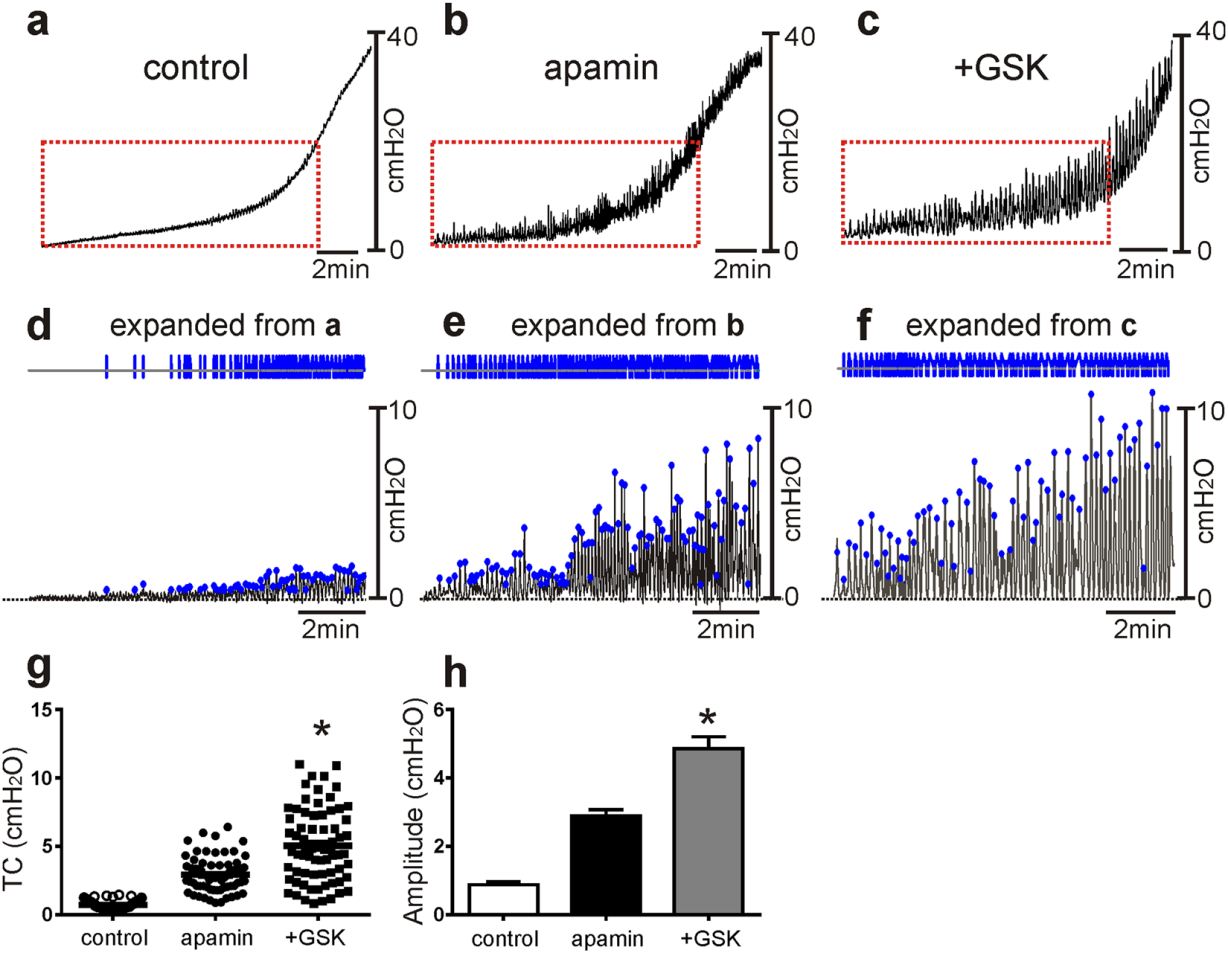
The effects of apamin and TRPV4 agonist on spontaneous activity of murine bladder using *ex vivo* preparation. *Ex vivo* pressure-volume response for control (**a**), after apamin application (**b**) and after addition of GSK in the presence of apamin (**c**). Expanded time scales with adjustment of baseline under control (**d**), apamin (**e**) and apamin + GSK (**f**) from panels a–c (red box). Blue lines and dots denote detected responses to analyze the amplitude distribution (see panel g). Amplitude and frequency distribution of transient contractions (TC) in control, apamin, and apamin + GSK treatment were analyzed from data in panels d–f (**g**). Summarized amplitude (**h**) under control, after apamin and apamin + GSK application (n = 6). Horizontal dotted lines in panels d–f denote baseline (0 cm H_2_0). *P < 0.001 by ANOVA.

We also examined the pressure-volume relationships of intact bladders using *ex vivo* preparations to test the effects of GSK and apamin. In contrast to *in vivo* cystometry, e*x vivo* preparation exclude extrinsic neural regulation during filling, so this technique focuses on myogenic mechanisms regulating detrusor excitability during filling. Infusion of Krebs–Ringer bicarbonate buffer (KRB; 15 µl/min) initially induced a small increase in intravesical pressure (0.9 ± 0.1 cm H_2_O, n = 12) before pressure rose steeply to a threshold pressure (about 20 cm H_2_O) for voiding contractions (see Fig. 6 and Methods). GSK alone did not significantly affect the pressure-volume responses up to 20 cm H_2_O (n = 6, data not shown), but apamin (300 nM) increased transients contractions during filling (2.2 ± 0.3 cm H_2_O, n = 6, *P* < 0.001 compared with control, Fig. 6a,b,d,e,g,h). After apamin, GSK increased the amplitude (to 5.1 ± 0.3 cm H_2_O, *P* < 0.001, n = 6) of the transient contractions (Fig. 6c,f–h). These effects were significant (p < 0.0001) by ANOVA.

**Figure 7:**
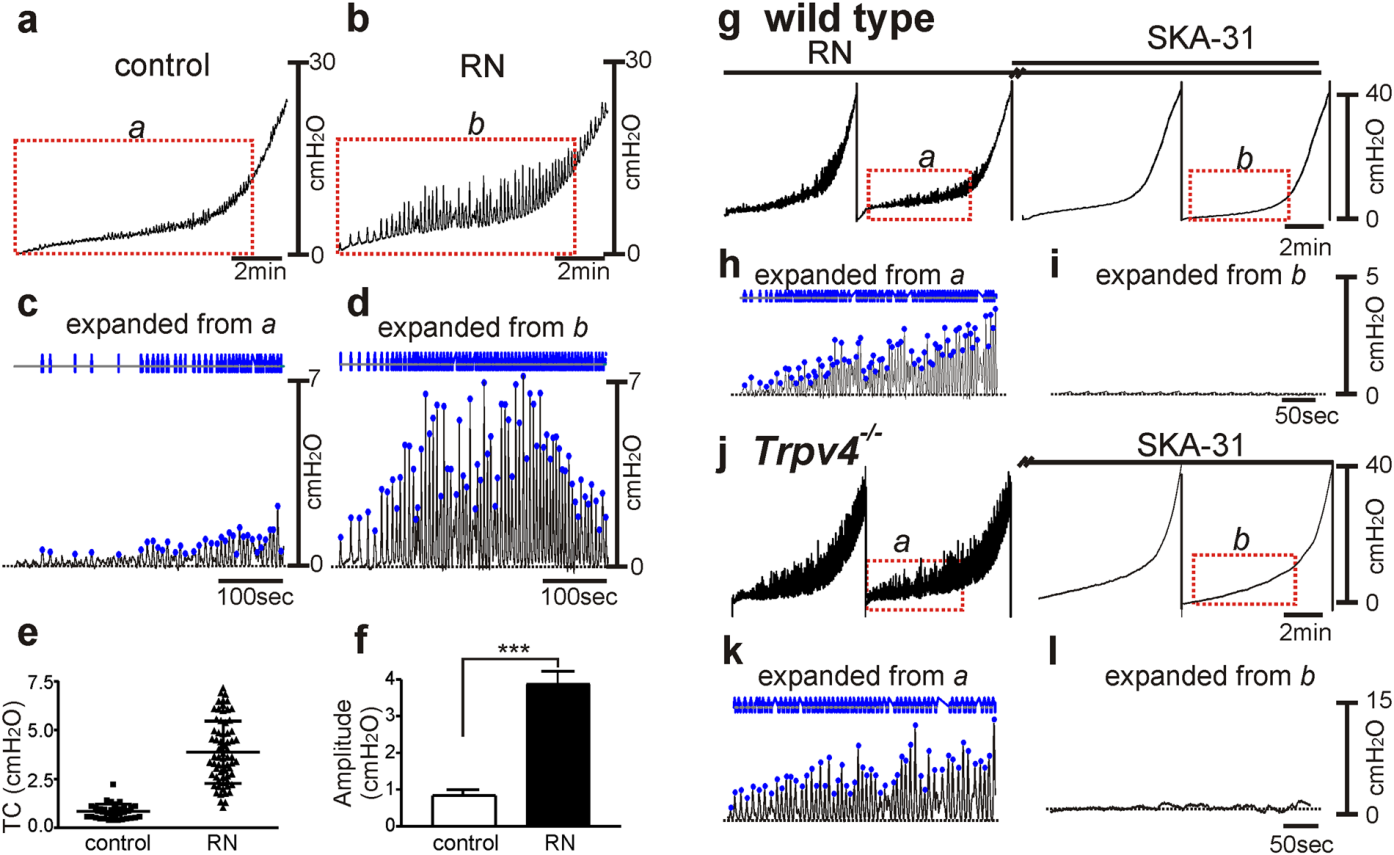
The effects of TRPV4 antagonist and SK channel activator in wild type and *Trpv4* ^−/−^ bladder on *ex vivo* preparation. Representative *ex vivo* pressure-volume curves in control (**a**) and RN-1734 (**b**). Expanded time scales with adjustment of baseline from red box in (**a** and **b**) (**c**,**d**). Blue lines and dots denote detected transient contractions (TC). Frequency and amplitude distribution of TC in control and after RN-1734 (RN) were analyzed by data in panels c and d (e). Summarized amplitude (**f**) in control and after RN- application (n = 6). Horizontal dotted lines in panels c and d denote baseline (0 cm H_2_0). Representative *ex vivo* pressure-volume curves in RN-1734 (**g**) and continuous application of SKA-31 (10 μM) in wild type animal. Expanded time scales after baseline adjustment under RN-1734 only (**h**) and combination with SKA-31 (**i**) from panel g red box. Gap (//) denotes 10 min incubation. (**j**) Representative *ex vivo* pressure response curve in control and after application of SKA-31 (10 μM) in *Trpv4* ^−/−^ animal. Expanded time scales after baseline adjustment under control (**k**) after SKA-31 (**i**) from panel **j** (red box). Gap (//) denotes 10 min incubation. ***P < 0.001, **P < 0.01.

TRPV4 antagonist, RN-1734 increased the transient contractions from 0.8 ± 0.1 cm H_2_O to 3.8 ± 0.4 cm H_2_O (n = 6, *P* < 0.001) during the filling phase (Fig. 7a–f). SKA (10 µM), in the presence of RN-1734, decreased the amplitude of transient contractions during filling (Fig. 7g–i). We also tested pressure responses in *Trpv4* ^−/−^ mice, and responses were similar in bladders of these mice as in presence of the TRPV4 antagonist (Fig. 7j,k). SKA-31 (10 µM) abolished the transient contractions during filling (Fig. 7j,l).

### Protein-protein interaction between TRPV4 and SK3 channels by western blot & Proximity Ligation Assay

**Figure 8:**
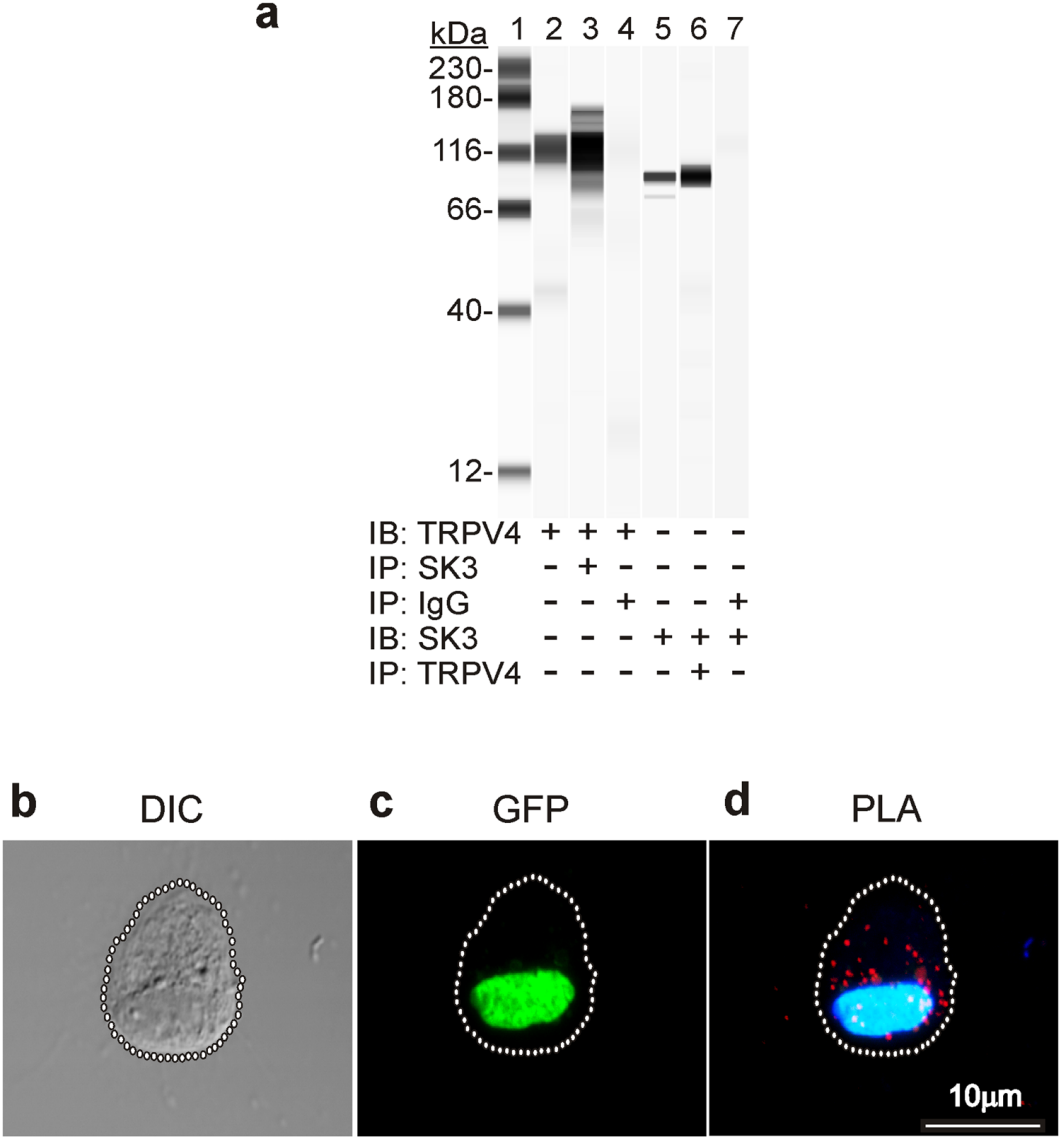
Co-immunoprecipitation and proximity ligation assay of TRPV4 and SK3 proteins. Immunoblotting (IB) for TRPV4 or SK3 in SK3 or TRPV4 immunoprecipitates (IP) of detrusor membrane fraction (n = 4, **a**). Lane 1, protein standards; lane 2, IB for TRPV4 in detrusor membrane fraction (1 μg); lane 3 IB for TRPV4 in SK3 IP; lane 4, IB for TRPV4 in non-immune rabbit IgG IP of detrusor membrane fraction; lane 5, IB for SK3 in detrusor membrane fraction (1 μg); lane 6, IB for SK3 in TRPV4 IP; lane 7, IB for SK3 in non-immune rabbit IgG IP of detrusor membrane fraction (n = 4, **b**). Isolated detrusor PDGFRα^+^ cell morphology visualized by DIC microscopy (**c**). The same cell verified as PDGFRα^+^ by GFP expression. PLA analysis of the same cell using goat anti-TRPV4 and rabbit anti-SK3 antibodies (**d**).

The specificity of the TRPV4 and SK3 antibodies were confirmed by immunoblots of detrusor membrane fractions (lane 2&5; Fig. 8a). TRPV4 was detected in the immunoblots of detrusor homogenates immunoprecipitated with the SK3 antibody (lane 3; Fig. 8a). The reverse co-immunoprecipitation (lane 6) shows that SK3 was detected in the immunoblots of detrusor homogenates immunoprecipitated with TRPV4 antibody. TRPV4 and SK3 were not detected in immunoblots of the detrusor membrane fraction incubated with non-immune rabbit IgG. These results suggest that there are protein-protein interactions between TRPV4 and SK3. Interactions between TRPV4 and SK3 in PDGFRα^+^ cells were investigated further by proximity ligation assays (PLA). PDGFRα^+^ cells were distinguished by the expression of eGFP (Fig. 8b,c). Incubation of the isolated detrusor PDGFRα^+^ cells with goat anti-TRPV4 and rabbit anti-SK3 antibodies followed by PLA detection show several sites of interaction present in these cells (Fig. 8d).

## Discussion

This study showed that expression of TRPV4 in detrusor smooth muscle tissues is due primarily to expression in PDGFRα^+^ cells. Patch clamp experiments confirmed that the TRPV4 conductance is functional in PDGFRα^+^ cells, because the TRPV4 agonist, GSK1016790A, activated inward current in these cells and little or no current in SMCs. Following the initial inward current, GSK also activated an outward current in PDGFRα^+^ cells when the cells were held positive to *E*_K_, but not when cells were held at *E*_*K*_. The outward current was due to activation of a conductance with properties consistent with it being due to SK channels, and UCL blocked the outward current activated by GSK. When the SK channels were blocked or when cells were held at *E*_*K*_, GSK activated only inward current. TRPV4 antagonists blocked both the inward and outward currents activated by GSK. TRPV4 has mechanosensitive properties^14^, and we hypothesized that detrusor excitability might be stabilized by activation of TRPV4 and subsequent activation of SK channels during bladder filling. The linkage between activation of TRPV4 and SK channels may be enhanced by protein-protein interactions between these channels, such that Ca^2+^ influx via TRPV4 channels activates SK channels. The functional relationship between TRPV4 channels and SK channels was verified in contractile and *ex vivo* bladder experiments. Inhibition of SK channels resulted in decreased compliance and increased transient contractions during filling. The same results were observed when TRPV4 channels were blocked or deactivated genetically, and an SK channel agonist reversed these effects. These findings describe a novel myogenic mechanism for controlling detrusor excitability during bladder filling and provide a rationale for the design of new therapies for overactive bladder.

TRPV4 channels (aka OTRPC4, VRL-2, VR-OAC, and TRP12) were first identified as a conductance activated by osmotic cell swelling^30–33^. Since this initial report, the properties of TRPV4 channels have been characterized extensively, and hypo-osmotic stress, mechanical stretch, temperature (30–43 °C) and phorbol ester derivatives are known to be modulators of TRPV4 activation^19–22,34–37^. TRPV4 channels have been linked to many cellular functions in various tissues throughout the body. For example, in vascular endothelium TRPV4 channels are linked to activation of IK and SK channels and dilation of resistance arteries^38,39^. These data suggested that Ca^2+^ influx through TRPV channels can directly regulate Ca^2+^-dependent K^+^ channels. Recently, we discovered cells distinguished by PDGFRα immunolabeling in detrusor muscles. These cells expressed SK3 channels but did not display functional voltage-dependent Ca^2+^ channels^5^. Thus, the present study sought to investigate a Ca^2+^ entry mechanism that might be linked to regulation of SK3 channels. With a pipette solution containing Cs^+^ to replace K^+^, inward current was activated by a TRPV4 agonist. However, when cells were dialyzed with a K^+^-rich solution, brief inward currents were followed by sustained outward current (e.g. holding potential −60 mV). TRPV4 channels are relatively Ca^2+^ selective with a *P*_Ca_/*P*_Na_ permeability ratio of about 6^29,32^. In our experiments, removal of Ca^2+^ from the external solution did not abolish the activation of inward current by GSK, but the outward current phase was completely blocked under these conditions. When cells were exposed to Na^+^-free and Ca^2+^-free solution, the inward and outward currents activated by GSK were completely abolished. These data are consistent with the idea that Ca^2+^ entry through TRPV4 channels (i.e. the inward current in response to GSK) activates outward current carried by SK3 channels.

Protein-protein interactions between TRPV4 and SK3 channels have been reported in rat mesenteric endothelial cells previously using co-immunoprecipitation and double immunolabeling methods^38^. This interactions between these proteins also appears to exist in detrusor muscles. However, it is possible that associations between proteins could develop in cell homogenates and involve proteins that are not naturally associated *in situ*. Therefore, we performed proximity ligation assays and found close associations between TRPV4 and SK3 channels in intact PDGFRα^+^ cells. Ca^2+^ concentrations at the intracellular mouths of activated TRPV4 channels may be far higher than in the greater cytoplasm, as shown by the development of sparklets and spatial decay of these events when TRPV4 channels are opened^40^. Thus, close associations between TRPV4 and SK3 may serve to directly regulate the open probability of SK3 channels.

Evidence for direct electrical coupling between PDGFRα^+^ cells and SMCs, as occurs in the gastrointestinal tract^41^, includes: (i) PDGFRα^+^ cells have high expression of SK3 channels and activation of these channels results in outward current and hyperpolarization of cells; SMCs have very low expression of SK channels and display no outward current or hyperpolarization response to SK channel activation at physiological holding potentials (−40 mV)^5^. (ii) Activation of SK conductance in intact detrusor muscles causes hyperpolarization and relaxation^5^. (iii) Blocking SK channels with apamin or genetically deactivating SK3 channels cause enhanced detrusor contractions^8,10^. (iv) P2Y1 receptors are expressed by PDGFRα^+^ cells and responses of these cells to purines include activation of SK channels; SMCs express only P2X-like inward currents in response to purines^6^. An inhibitory response to purines is manifest in whole detrusor muscles and is blocked by P2Y1 antagonists and in *P2ry1* ^−/−^ mice^6^. (v) TRPV4 is expressed by PDGFRα^+^ cells and an agonist for these channels activates inward current and is secondarily coupled to activation of SK currents (current study). vi) SMCs have low expression of TRPV4, no response is activated in SMCs by the TRPV4 agonist, and detrusor muscles relaxed in a concentration-dependent manner to TRPV4 agonists unless SK channels were blocked by apamin (current study). For electrical responses transduced by PDGFRα^+^ cells to affect detrusor contractility, low resistance electrical coupling must exist between PDGFRα^+^ cells and SMCs.

A functional role for TRPV4 channels in regulating contractile behavior of the urinary bladder has been investigated previously with drugs to activate or block TRPV4 channels in wild-type mice and global *Trpv4* ^−/−^ mice^15,16^. Previous studies reported that GSK enhanced contractions in strips of muscle with the urothelium intact or removed, and these responses were attributed to effects on SMCs^15,42^. We were unable to reproduce these results in the many experiments we performed using the same experimental conditions on detrusor muscles from the same strain of mice (C57BL/6). In fact, GSK caused concentration-dependent inhibition of contractions across the range of concentrations tested in 37 of 44 detrusor smooth muscle strips. In a small number of muscles (7 of 44) high concentrations of GSK caused slight restoration of contractions. The reasons for the differences in results are unclear, but with little or no TRPV4 expression in SMCs, no current responses activated by GSK in SMCs, molecular and functional expression of TRPV4 channels in PDGFRα^+^ cells, and linkage between Ca^2+^ entry via TRPV4 and activation of SK3 channels in PDGFRα^+^ cells, it is difficult to understand how GSK could elicit contractile responses in intact muscles. We also found that when muscles were treated with apamin to block SK channels, GSK activated small contractile responses. Contractions in the presence of apamin were likely due to the inward current and depolarization responses elicited in PDGFRα^+^ cells because expression of TRPV4 is extremely low in SMCs, and accordingly these cells displayed no activation of current in response to GSK. Finally, we also examined the effects of the TRPV4 antagonist (RN-1734). This compound increased detrusor contractions and the SK channel agonist abolished these responses. Taken together, these data support the concept that TRPV4 channels form a source of Ca^2+^ entry in PDGFRα^+^ cells that is linked directly to activation of SK channels. We detected no inward current activation attributable to TRPV4 in SMCs in any of our experiments.

*Ex vivo* preparation experiments were used to test myogenic mechanisms involved in regulation of TCs. These studies showed that TRPV4/SK3 coupling in PDGFRα^+^ cells provides an important means of moderating detrusor excitability during bladder filling. Apamin enhanced the frequency and amplitude of contractions during filling, confirming the idea that SK channels regulate detrusor excitability. In the presence of apamin, GSK increased the amplitude of contractions, suggesting that the inward currents activated in PDGFRα^+^ cells by GSK (see Fig. 4) can exert a depolarizing effect on detrusor muscles in intact bladders in the absence of SK channel activation. Finally, RN-1734, an antagonist of TRPV4 channels, increased contractions during filling. The enhanced contractions when TRPV4 channels were blocked by RN-1734 were reversed by SKA-31, an agonist for SK channels. These findings show that TRPV4 channels are coupled to stabilization of detrusor excitability through activation of SK channels. This is a previously unrecognized regulatory mechanism provided by PDGFRα^+^ cells and contributing to the integrated responses of detrusor muscles.

The role of TRPV4 was also tested by performing *ex vivo* preparation experiments on bladders from *Trpv4* ^−/−^ mice. In a previous study *Trpv4* ^−/−^mice displayed decreased frequency in voiding contractions as measured by *in vivo* cystometry and voiding spot analysis^16,17^. Also apparent in the responses *Trpv4* ^−/−^ mice, but not commented upon in previous studies, was a significant increase in non-voiding contractions^16,17^. The decrease in voiding contractions was thought to be due to reduced TRPV4 in the urothelium and thus an altered sensory phenotype^16^. In the urothelium TRPV4 expression has been postulated to regulate stretch-mediated release of ATP, a primary sensory mediator regulating the output of bladder afferent neurons^16,43^. However, the hypothesized urothelial functions of TRPV4 channels cannot explain the increase in non-voiding contractions observed in *Trpv4* ^−/−^ mice. In addition to the proposed sensory role of TRPV4 in the bladder, our data suggest a novel role for these channels in myogenic regulation of detrusor excitability. TRPV4 channels are mechanosensitive and can be activated by stretch of cells^29,30,32,36^. Thus our findings suggest the following myogenic mechanism: as detrusor smooth muscle tissues are stretched during bladder filling, activation of TRPV4 channels provides Ca^2+^ entry into PDGFRα^+^ cells; the rise in [Ca^2+^]_i_ (possibly localized near the cytoplasmic mouths of TRPV4 channels) activates SK3 channels that are highly expressed in PDGFRα^+^ cells^5^; enhanced outward current through SK3 channels causes hyperpolarization which conducts to electrically-coupled SMCs, restraining the excitability of these cells and reducing the tendency of SMCs to generate transient contractions. This non-neural, moment-to-moment mechanism for controlling excitability of detrusor SMCs and increasing bladder compliance is a novel physiological regulatory feature of normal bladders. Loss-of-function of this mechanism in genetic models such as *Trpv4* ^−/−^ mice or after pharmacological block of TRPV4 channels or SK3 channels leads to enhanced transient contractions and symptoms experienced in overactive bladder syndrome. Finding a means to selectively regulate this pathway may provide novel therapeutic approaches to controlling overactive bladder.

## Methods

### Animal preparation and Cell Isolation of detrusor PDGFRα^+^ cells and SMCs

All experimental procedures were conducted in accordance with the National Institutes of Health *Guide for the Care and Use of Laboratory Animals* and the animal use protocol, reviewed and approved by the Institutional Animal Use and Care Committee at the University of Nevada. C57BL/6, *Pdgfra* ^*tm11*(*EGFP*)*S*^ *°* ^*r*^/J (both purchased from Jackson Laboratory, Bar Harbor, ME, USA), smMHC/Cre/eGFP (donated by Dr. Michael Kotlikoff, Cornell University), and *Trpv4* ^−/−^ mice (Donated by Dr. Kevin Thorneloe, GlaxoSmithKline LLC)^15^ were used. We used male only (8 to 12 weeks old) to exclude the effect of female steroid hormone on the ion channel expression. Dissection of detrusor smooth muscle was performed as previously described^5^. Briefly, Detrusor single SMCs and PDGFRα^+^ cells were isolated from detrusor tissues by enzymatic digestion using a combination of papain and collagenase^5^. Single SMCs and PDGFRα^+^ cells were used for patch clamp experiments within 12 h of isolation at 30 °C.

### Cell purification and transcriptional analysis

Fluorescence-activated cell sorting (FACS; Becton Dickinson FACSAria using blue laser 488 nm and GFP emission detector; 530/30 nm) was used to purify PDGFRα^+^ cells and eGFP/SMCs. Tests for cell purity were described previously^5^. According to the manufacturer’s instructions, total RNA was isolated from PDGFRα^+^ cells and eGFP/SMCs using illustra RNAspin Mini RNA Isolation kit (GE Healthcare, Little Chalfont, UK), and first-strand cDNA was synthesized using SuperScript III (Invitrogen, Carlsbad, CA, USA). PCR products with specific primers (Supplementary Table S1) using Go-Taq Green Master Mix (Promega Corp., Madison, WI, USA) were analyzed on 2% agarose gels and visualized by ethidium bromide. The same primers as PCR using Fast Syber green chemistry (Applied Biosystems, Foster City, CA, USA) on the 7900HT Real Time PCR System (Applied Biosysytems) were used for Quantitative PCR (qPCR). Standard curves were generated by PCR Regression analysis of the mean values of three multiplex qPCRs for the log10 diluted cDNA. To give transcriptional quantification of each gene relative to the endogenous *Gapdh* standard after log transformation of the corresponding raw data, unknown amounts of messenger RNA (mRNA) were plotted relative to the standard curve for each set of primers which graphically plotted using Microsoft Excel and Graphpad Prism (v. 3.0, Graphpad Software Inc., San Diego, CA, USA) softwares.

### Electrophysiological recordings

When filled with the pipette solution, tip resistances were 3-4 MΩs. Whole cell configuration was achieved in CaPSS bath solution (mM): NaCl 135, KCl 5, MgCl_2_ 1.2, CaCl_2_ 2, Glucose 10, HEPES 10, pH 7.4 with Tris-base. The MnPSS bath solution (also called Ca-0 solution) was replaced with equimolar Mn^2+^ in CaPSS solution. The Ca^2+^−0/Na^+^−0 solution was replaced with equimolar Mn^2+^ and NMDG^+^ in CaPSS solution. The K^+^-rich/Cs^+^-rich pipette solution contained (mM): KCl/CsCl 135, CaCl_2_ 0.012, MgATP 3, Na_2_GTP 0.1, Creatine phosphate disodium 2.5, EGTA 0.1, Glucose 10, HEPES 10, pH 7.2 with Tris-base, respectively. Whole cell voltage- and current-clamp techniques were performed. Cells were placed in a 0.5 ml chamber mounted on an inverted microscope (Nikon, Japan). PDGFRα^+^ cells were identified by the fluorescence of eGFP in nuclei^5^. SMCs were easily identified by their morphology. An Axopatch 200B amplifier with a CV-4 headstage (Molecular Devices, Sunnyvale, CA, USA) was used. For mechanical stretch experiments, a gigaohm seal was formed with one pipette for recording whole cell currents, and the second pipette was cell-attached to a micromanipulator and used for stretching cells. All data were analyzed using pCLAMP software (Molecular devices, Sunnyvale, CA, USA) and Graphpad Prism. All recordings were made at ~30 °C.

### Isometric Force Measurement

Contractions of strips of murine detrusor muscles were measured by standard organ bath techniques^6^. Briefly, muscle strips were attached to a fixed mount and an isometric force transducer (Fort 10, WPI, Sarasota, FL, USA). Muscles were immersed in organ baths perfused with oxygenated (95% O_2_ and 5% CO_2_) Krebs–Ringer bicarbonate buffer (KRB) solution and bath temperature was maintained at 37.5 ± 0.5 °C. The muscles were exposed to different concentrations of TRPV4 agonist, GSK (GSK1016790A) in the absence and presence of apamin and allowed to respond for at least 5 min to each concentration when stable responses were obtained. TRPV4 antagonist and SK channel agonist and antagonist were also tested. The effects of GSK were analyzed by calculating the average amplitude, frequency, and AUC (the area under curve) during control conditions and 5 min after addition of GSK. Contractile activity was recorded by computer running LabChart8 (AD Instruments, Colorado Springs, CO, USA) and measurements of peak amplitude and sustained amplitude were obtained.

### *Ex vivo* preparation

Bladders were removed from animals and the ureters were ligated closed at the vesicoureteric junctions. A single catheter was placed in the urethral opening to fill the bladder and simultaneously measure the pressure. Intravesical pressure was recorded with reference to the air (i.e. atmospheric pressure = 0) by a water-filled pressure transducer placed level with the bladder and connected to a quad-bridge amplifier (AD Instruments). The infusion rate was 25 µl/min by a Genie Touch automated syringe pump (Kent Scientific) with KRB solution at 37 °C. Filling was stopped when the bladder pressure reached 40–45 cm/H_2_O to avoid over-distention which can induce permanent tissue damage. The effects of TRPV4 channel agonist and antagonist and SK channel agonist and antagonist were tested. *Trpv4* ^−/−^ mice were also used for *ex vivo* volume-pressure evaluation. *Ex vivo* data were analyzed by Clampfit (Molecular Devices) with baseline adjustment to examine the frequency and amplitude of transient contractions occurring during the filling phase.

### Co-immunoprecipitation

Detrusor smooth muscles from two mice were placed into 0.5 ml of ice cold lysis buffer (mM; 50 Tris HCl pH 8.0, 60 ß-glycerophosphate, 100 NaF, 2 EGTA, 25 Na-pyrophosphate, 1 DTT, 0.001% antifoam (Sigma) and protease inhibitor tablet (Roche, Indianapolis, IA, USA), and homogenized using a Bullet Blender (stainless steel bead, speed 7, 5 min) (Next Advance, Averill Park, NY, USA). The homogenates were centrifuged at 16,000 × g at 4 °C for 10 min, and the supernatants were then centrifuged at 100,000 × g at 4 °C for 1 hour. the 100,000 × g pellet (membrane fraction) was resuspended into lysis buffer 0.1% SDS, and analyzed for protein content with the Bradford assay. For the co-immunoprecipitation, 500 µg of pellet protein was pre-cleared with 25 µl of protein A/G-Dynabeads suspension (Thermo Fisher Scientific, Waltham, MA, USA) (rotating for 30 min at 4 °C). The rabbit TRPV4 (10 µg) (Abcam, Cambridge, MA, USA) or the rabbit SK3 antibody (10 µg) (Alomone Labs, Jeruselem, Israel) were each crosslinked to 25 μl of protein A/G-Dynabeads with DSS (disuccinimidyl suberate), using the Pierce Crosslink Immuno-precipitation Kit (Thermo Fisher Scientific, Waltham, MA, USA). The pellet protein samples were incubated with the protein A/G-Dynabeads suspension for 2 hours with rotation at 4 °C. The beads were placed on the magnet, the supernatants (flow-through) removed, and the beads washed three times with 200 µl lysis buffer. Immunoprecipitated proteins were eluted with low pH elution buffer, and then neutralized with the high pH buffer.

### Automated capillary electrophoresis and chemiluminescent western blotting

Analysis of the IP eluates was performed using a Wes instrument (ProteinSimple (San Jose, CA, USA). The samples were mixed with fluorescent 5X Master Mix and incubated at 95 °C for 5 min. The samples were loaded into the Wes plate (Wes 12–230 kDa Pre-filled Plates with Split Buffer) along with biotinylated protein ladder, blocking reagent, primary antibodies, ProteinSimple HRP-conjugated anti-rabbit secondary antibody, luminol-peroxide, and washing buffer. The plates and capillary cartridges were loaded into the Wes for electrophoresis and chemiluminescence immunodetection imaging by a CCD camera using default settings: electrophoresis, 375 volts 25 min; blocking, 5 min; primary antibody, 30 min; secondary antibody, 30 min; camera exposure times, 1 sec to 120 sec. Compass software (ProteinSimple) was used to acquire and analyze the data, and to generate gel images and chemiluminescence signal intensity values. The digital lane views (bitmaps) of the immunodetected protein bands were generated by Compass software, with each lane corresponding to an individual capillary tube. The protein immunodetection figure was created from the digitized data using Corel PhotoPaint and Corel Draw × 4 (Corel Corp., Ottawa, Ontario, Canada).

### Proximity Ligation Assay (PLA)

Enzymatically isolated detrusor PDGFRα^+^ cells were incubated overnight on slides coated with murine collagen (2.5 mg/ml, BD Falcon, Franklin Lakes, NJ, USA), fixed in 4% paraformaldehyde for 4 min, and then permeabilized and blocked with PBS containing 0.2% tween-20 and 1% bovine serum albumin for 10 min at room temperature. PLA was performed following the manufacturer’s instructions (Duolink Detect, Olink Bioscience, Sweden). The fixed and permeabilized cells were incubated with goat anti-TRPV4 specific antibody (1:200 dilution) (Santa Cruz Biotechnology, Santa Cruz, CA, USA) for 1 hour at room temperature, washed 3 times with PBS and then incubated with the rabbit anti-SK3 antibody (1:200 dilution) (Alomone Labs, Jerusalem, Israel) for 1 hour at room temperature. The slides were washed 3 times with PBS and then incubated with the Duolink minus-anti rabbit IgG and plus-anti goat IgG secondary antibodies (1:5 dilution) at 37 °C for 1 hour, followed by the ligation and amplification reactions (Duolink detection kit red, ex.598/em.634). Finally, mounting medium with DAPI was used. The slides were examined using a LSM510 Meta (Zeiss, Jena, Germany) or Fluoview FV1000 confocal microscope (Olympus, Center Valley, PA, USA). Confocal micrographs are digital composites of the Z-series of scans (0.5 μm optical sections of 7 μm thick sections). Settings were fixed at the beginning of both acquisition and analysis steps and were unchanged. Final images were constructed using FV10-ASW 2.1 software (Olympus). In negative control, primary anti-TMEM16a and Myl9 (Santa Cruz Biotechnology Inc., Santa Cruz, CA, USA) antibodies were used.

### Drugs

All drugs and reagents including GSK1016790A, HC-067047, RN-1734, SKA-31, UCL1684 and 4α-PDD were purchased from Sigma (Sigma-Aldrich, Co., Louis, MO, USA). Apamin was purchased from Santa Cruz Biotech (Santa Cruz Biotechnology, Inc., Dallas, Texas, USA). Dimethyl sulfoxide (DMSO) was used to make stock solutions. Final concentration of DMSO in the bath solution was less than 0.01%.

### Statistical analyses

All data were expressed as means ± S.E.M. All statistical analyses were performed using Graphpad Prism. Student’s paired, unpaired *t* test or one-way ANOVA were used to compare groups of data and differences were considered to be significant at *P* < 0.05.

## Electronic supplementary material

supplementary data
