# Supplementary material for: Premature contractions of the bladder are suppressed by interactions between TRPV4 and SK3 channels in murine detrusor PDGFRα+ cells
Source: Sci Rep. 2017 Sep 25;7:12245. doi: 10.1038/s41598-017-12561-7 (PMC5613012; doi:10.1038/s41598-017-12561-7)
Supplement: Supplementary file 1 — supplementary data [file 41598_2017_12561_MOESM1_ESM.pdf]

Premature contractions of the bladder are suppressed by interactions between TRPV4 and SK3 channels in murine detrusor PDGFRα⁺ cells

Haeyeong Lee[1*], Byoung H. Koh[1], Lauren E. Peri[1], Robert D. Corrigan[1], Hyun-Tai Lee[1], Nikita E. George[1], Bhupal P. Bhetwal[1], Yeming Xie[1], Brian A. Perrino[1], Toby C. Chai[2], Kenton M. Sanders[1], Sang Don Koh[1]

Supplementary Fig. S1: The effect of GSK on the detrusor smooth muscle cells.

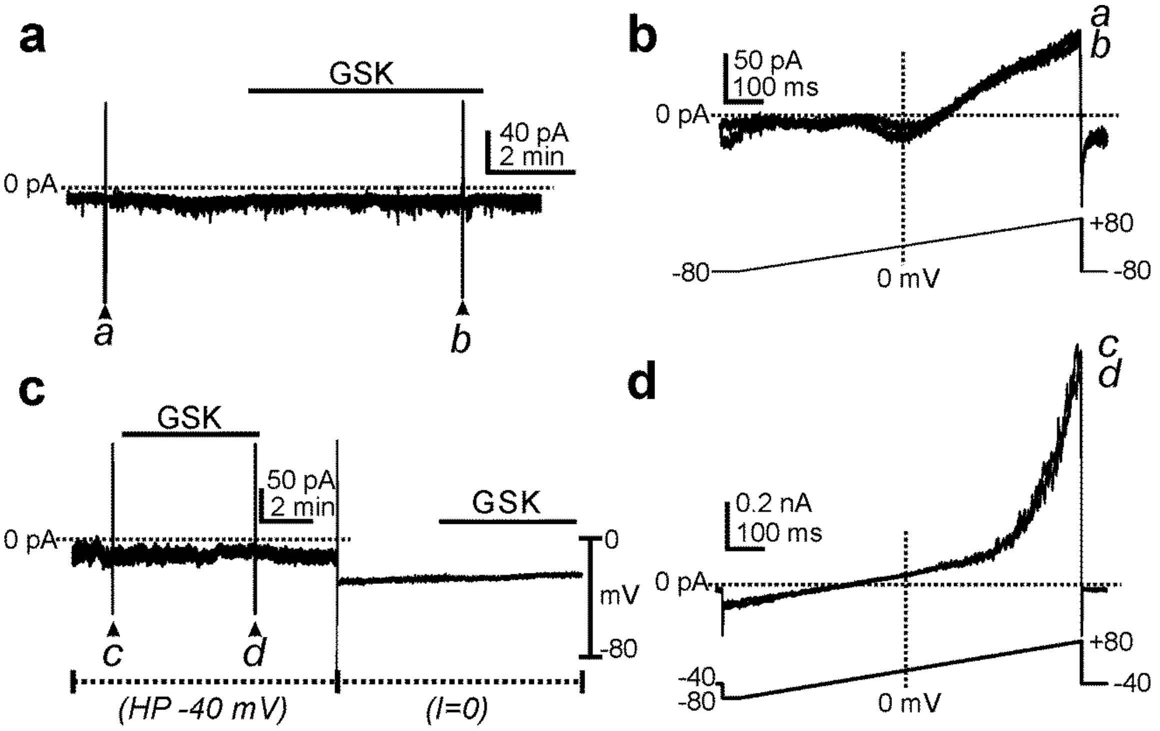

Cells were dialyzed with Cs^{+}-rich (**a, b**) or K^{+}-rich (**c, d**) solutions. GSK (100 nM) did not evoke any current in smooth muscle cells with a holding potential of -80 mV (**a**). Expanded time scales from panel **a** during ramp depolarization (see inset) before (*a)* and after *(b)* GSK. GSK did not activate the outward current at a holding potential of -40 mV and had no effect on the membrane potential under current-clamp mode (*I=0,* **c**). Expanded time scales from panel **c** during ramp depolarization before (*c)* and after *(d)* GSK (**d**).

Supplementary Fig. S2: The property of TRPV4 channels in detrusor PDGFRα$^+$ cells.

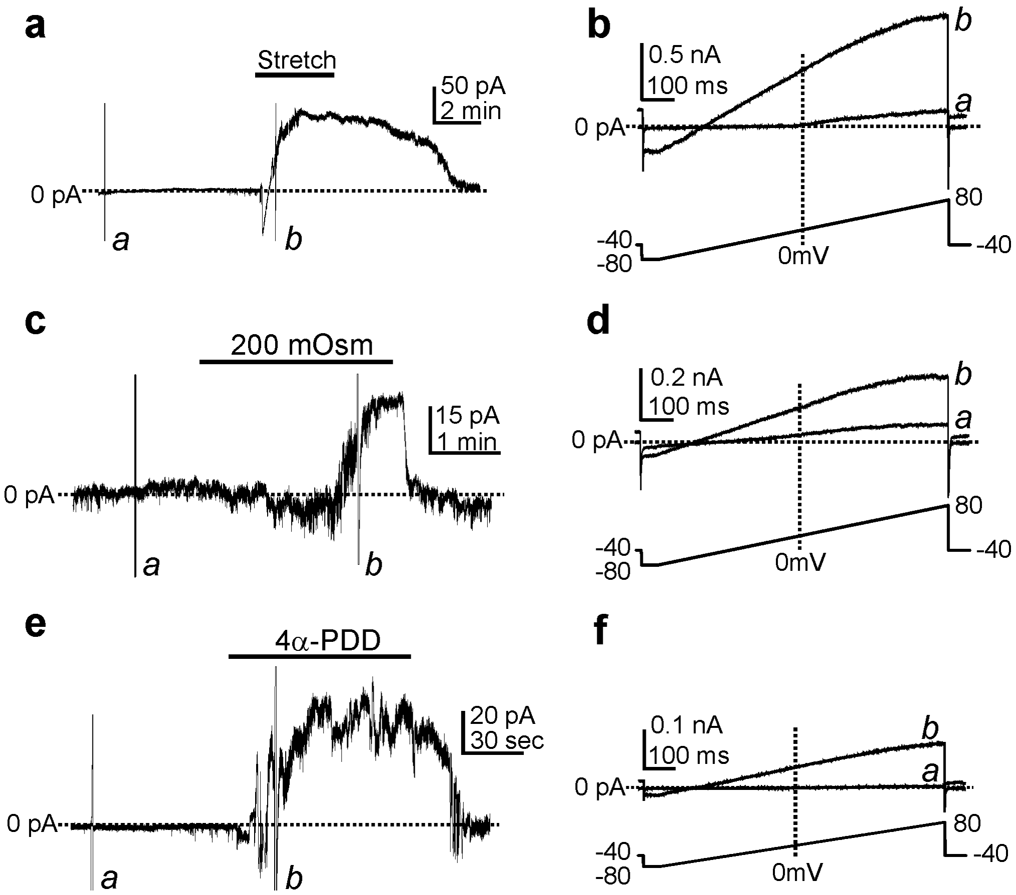

The effects of mechanical stretch, hypo-osmotic stress and 4α-Phorbol 12,13-didecanoate (4-α-PDD) on generation of outward currents were tested in PDGFRα$^+$ cells dialyzed with K^+-rich solutions at a holding potential of -40 mV. Cell elongation (1-2 µm) induced initial inward current followed by the outward current at a holding potential of -40 mV (**a**). Expanded time scales (**b)** from panel **a** during ramp depolarization before (*a)* and after *(b)* cell elongation. Hypo-osmotic stress and 4α-PDD exposure induced inward currents followed by outward currents (**c** & **e**, respectively). Expanded time scales (**d** & **f**) showed ramp depolarizations from panel **c** and **e** before (*a)*, after *(b)* application of 200 mOsm solution and 4α-PDD*.*

Supplementary Table S1. Primer Sources

Gene Name	Sequence (Sense primer on top)	Accession #
Gapdh	GCCGATGCCCCCATGTTTGTGA<br>GGGTGGCAGTGATGGCATGGAC	NM_008084
Pdgfra	ATGACAGCAGGCAGGGCTTCAACG<br>CGGCACAGGTCACCACGATCGTTT	NM_011058
Myh11	CAGCTGGAAGAGGCAGAGGAGG<br>AACAAATGAAGCCTCGTTTCCTCTC	NM_013607
Kit	CGCCTGCCGAAATGTATGACG<br>GGTTCTCTGGGTTGGGGTTGC	NM_021099
Uchl1	CGATGGAGATTAACCCCGAGATG<br>TTTTCATGCTGGGCCGTGAG	NM_011670
Trpc1	CTGTGGATTATTGGGATGATTTGGTCA<br>CACTTTGAGGGCAAAGGTTGCCAA	NM_011643
Trpc2	CTCCTCCTGGCTGGGCTTG<br>GGTGAAGCTGAGCATGCTGGTG	NM_011644
Trpc3	CAGCCAACACGATATCAGATAAT<br>AAGTTCATAACGAAGGCTGGAGATATC	NM_019510
Trpc4	CCATGACTGGTGGAATCTAAT<br>GGTGCCACATGTCCCATGATT	NM_016984
Trpc5	CATCTCCCTGGTAGTGCTGCTG<br>CACCTTCATCAAAGTAACTCATCCAGAG	NM_009428
Trpc6	GTGGTGCTTGCTGTTGTCATTG<br>CTTCAAATCTGTCAGCTGCATTCATGAC	NM_013838
Trpc7	CTCATAATGAGAATCAAGATGTGCCTC<br>CAGAATTCCTCATGCCAGCCT	NM_012035
Trpm1	CGTGGGTTGAAGTTGTTCCTTAGC<br>TCGTTGATTTCCTCCAATCTCATTGAC	NM_018752

Trpm2	CTTCTGGAACAAACTGGACGTTGG<br>GAAGATGTGCATGAGACGGAGAC	NM_138301
Trpm3	GCTGACCAGATAGACCGTAAGC<br>GACAATCCATGCTCCTGTCTTGC	NM_001035239
Trpm4	GGAGCAGGTCCTCCTGGGACT<br>GGCACCCCAGAAGTCCGACG	NM_175130
Trpm5	CAACTGGAACAAGTGTGACATGGTG<br>GCAAAGATGTGGATGAGCCGAAGTG	NM_020277
Trpm6	CGGCTCATGGACTTCTTTGCTG<br>GGCTCAAAGACGATGTCACGAG	NM_153417
Trpm7	GTGCGTTTGCTAGACTTTCTAGCC<br>CAAGAGACCAAGATGGTTCTTCATGTG	NM_021450
Trpm8	GCTGGGAGTCTGCCGACCTT<br>GGGTGCTGAAGTGGGTGGAGA	NM_134252
Trpv1	GAGAGCAAGAACATCTGGAAGCTGC<br>GATGATGCCCACGTTGGTGTTCC	NM_001001445
Trpv2	GAGCTGGACTGGACTGCTAGAGTAC<br>TTCAGCACAGCCTTCATCAGGCAC	NM_011706
Trpv3	GAAGCGACTGAAGAAGCGCATCTTC<br>TAGGAGACCACGTTGATGTAGAAGG	NM_145099
Trpv4	GTGCTGGAGATCCTGGTGTACAAC<br>GTCAGCTTGTGCATGAGGAAGTCAG	NM_022017
Trpv5	AGACCAAGACTGGAACCAGCACG<br>TCCCAGAGCTCCTCTTTGCCG	NM_001007572
Trpv6	GAGCCGAGACGAGCAGAACCT<br>CCCATGGCTCCTCTCTGGTGC	NM_022413
